# Eating disorder symptoms and their associations with anthropometric and psychiatric polygenic scores

**DOI:** 10.1002/erv.2889

**Published:** 2022-02-17

**Authors:** Mohamed Abdulkadir, Christopher Hübel, Moritz Herle, Ruth J. F. Loos, Gerome Breen, Cynthia M. Bulik, Nadia Micali

**Affiliations:** 1 Department of Pediatrics Gynaecology and Obstetrics, Faculty of Medicine, University of Geneva, Geneva, Switzerland; 2 Department of Psychiatry, Faculty of Medicine, University of Geneva, Geneva, Switzerland; 3 Social, Genetic & Developmental Psychiatry Centre, Institute of Psychiatry, Psychology & Neuroscience, King’s College London, London, UK; 4 UK National Institute for Health Research (NIHR) Biomedical Research Centre for Mental Health, South London and Maudsley Hospital, London, UK; 5 National Centre for Register-based Research, Aarhus Business and Social Sciences, Aarhus University, Aarhus, Denmark; 6 Department of Medical Epidemiology and Biostatistics, Karolinska Institutet, Stockholm, Sweden; 7 Department of Biostatistics & Health Informatics, Institute of Psychiatry, Psychology & Neuroscience, King’s College London, London, UK; 8 Icahn School of Medicine at Mount Sinai, Charles Bronfman Institute for Personalized Medicine, New York, New York, USA; 9 Department of Psychiatry, University of North Carolina at Chapel Hill, Chapel Hill, NC, USA; 10 Department of Nutrition, University of North Carolina at Chapel Hill, Chapel Hill, NC, USA; 11 Great Ormond Street Institute of Child Health, University College London, London, UK

**Keywords:** anthropometric traits, Avon longitudinal study of parents and children (ALSPAC), metabolic traits, psychiatric traits

## Abstract

**Background:**

Eating disorder (ED) symptoms are prevalent in the general population, but their shared genetic underpinnings with psychiatric, metabolic, and anthropometric traits are not known. Here, we examined if polygenic scores (PGSs) of traits associated with anorexia nervosa are also associated with adolescent ED symptoms in the Avon Longitudinal Study of Parents and Children (ALSPAC).

**Methods:**

A total of 8654 participants with genotype data and at least one phenotypic measure were included from the ALSPAC study. We associated PGS from 25 traits (16 psychiatric, 4 metabolic, and 5 anthropometric) with eight ED symptoms, including behaviours such as fasting for weight loss and cognitions such as body dissatisfaction.

**Results:**

Higher attention deficit hyperactivity disorder PGS and lower educational attainment PGS were associated with fasting for weight loss. Higher insomnia PGS was associated with increased body dissatisfaction. We found no evidence of an association between metabolic trait PGS and any ED symptom. Fat-free mass, fat mass, and body fat percentage PGSs, were positively associated with binge eating, excessive exercise, fasting for weight loss, body dissatisfaction, and weight and shape concern.

**Conclusions:**

ED symptoms are genetically associated with psychiatric and anthropometric, but not with metabolic traits. Our findings provide insights for future genetic research investigating on why some individuals with ED symptoms progress to develop threshold EDs while others do not.

## 1 | INTRODUCTION

Eating disorder (ED) symptoms are prevalent (18%–22%) in the general population and include behavioural symptoms (e.g., binge eating, purging, and restrictive eating) and cognitive symptoms (e.g., body dissatisfaction, drive for thinness; Hayes et al., 2018; Micali, De Stavola, et al., 2015; Micali et al., 2017). Epidemiological studies suggest that ED symptoms are phenotypically associated with psychiatric traits (e.g., mood disorders, psychosocial impairment, and suicidal behaviour) and anthropometric traits (Chamay-Weber et al., 2005; Crow et al., 2008; Micali et al., 2017; Reed et al., 2017). Especially for anthropometric traits, recent evidence suggests a causal bidirectional relationship between ED symptoms and body mass index (BMI): higher BMI has an effect on increased ED symptoms and vice versa (Reed et al., 2017). Therefore, given the continuing rising prevalence of obesity world-wide (Jaacks et al., 2019), the prevalence of eating disorder symptoms may rise in tandem. From a public health perspective, it is therefore important to understand the aetiology of ED symptoms (Dinkler et al., 2019).

ED symptoms have been shown to be moderately heritable (*h*^2^ = 0.65, 95% confidence interval = 0.62–0.69; Dinkler et al., 2019). To date, most genome-wide association studies (GWASs; a genetic study in which genetic variants, across the entire genome, are tested for their association with a trait of interest) have focussed on anorexia nervosa (AN) caseness (Boraska et al., 2014; Duncan et al., 2017; Li et al., 2017; Watson et al., 2019) with little attention directed towards understanding the genetic architecture of symptoms that underlie ED (e.g., body dissatisfaction). Notable exceptions are two GWASs carried out by Boraska et al. (2012) and Wade et al. (2013) that focussed on ED symptoms such as drive for thinness, body dissatisfaction, and bulimia. The authors from both studies failed to detect a genome-wide significant locus, however, in the study led by Boraska et al. the author reports that the top suggestively associated single nucleotide polymorphisms (SNPs; single base-pair difference in the DNA sequence of among individuals in a population) had previously been associated with other psychiatric disorders; namely, major depressive disorder and schizophrenia (Amato et al., 2010; Aragam et al., 2011; Boraska et al., 2012). This lack of genome-wide significant signal in both studies was likely due to the typically small-to-modest effect sizes of the common SNPs that influence complex traits that require large sample sizes in the thousands for detection.

Polygenic score (PGS) analysis is a powerful method that leverages the small-to-modest effect sizes of common SNPs into a single continuous score that exceeds the explanatory power of single SNPs (International Schizophrenia Consortium et al., 2009). PGSs can be used to examine whether two traits are genetically related by associating the PGSs calculated for one trait with another trait. Our group (Abdulkadir et al., 2020) and others (Nagata et al., 2019; Robinson et al., 2020; Solmi et al., 2019) have successfully applied this method and reported that a BMI PGS and a schizophrenia PGS are associated with ED symptoms (e.g., binge eating, fasting for weight loss, and body dissatisfaction) in the general population. Furthermore, in previous work, we demonstrated that the association between a BMI PGS and ED symptoms was mediated through measured BMI; we found that an increase in the BMI PGS corresponded to an increase in zBMI scores at age 11 years, which in turn led to an increased probability of endorsing ED symptoms during puberty (Abdulkadir et al., 2020). However, a broader evaluation of PGSs indexing psychiatric and anthropometric/metabolic traits is warranted, as GWASs on AN reported several significant genetic correlations with psychiatric, metabolic, and anthropometric traits (Duncan et al., 2017; Hübel et al., 2019; Watson et al., 2019); in other words, ED symptoms could also be genetically correlated with psychiatric, metabolic, and anthropometric traits. Furthermore, several psychiatric traits are known to be associated with BMI suggesting possible mediation effects between psychiatric PGS and ED symptoms through BMI (Benson et al., 2018; Hübel et al., 2019; McCrea et al., 2012).

The aim of this study was to investigate whether PGSs of psychiatric, metabolic, and anthropometric traits that are genetically associated with AN are also associated with behavioural and cognitive ED symptoms and whether these associations are possibly mediated by BMI in the Avon Longitudinal Study of Parents and Children (ALSPAC). ALSPAC is a general population cohort which allows for separating out ED symptoms from eating disorders as such, given that by definition those that have ED symptoms in a clinical sample also have a disorder, whilst this is mostly not the case in a general population cohort. The ALSPAC sample is also ideal for this type of study as it has longitudinal information on ED symptoms. We hypothesised that like AN (Watson et al., 2019), behavioural and cognitive ED symptoms would be associated with anthropometric, metabolic, and psychiatric PGSs.

## 2 | METHODS

### 2.1 | Participants

The ALSPAC study is an ongoing population-based birth cohort study of mothers and their children (that were born between 1 April 1991 and 31 December 1992) residing in the southwest of England (UK) (Boyd et al., 2013; Fraser et al., 2013; Golding, 2004; Golding et al., 2001; Northstone et al., 2019). From the 14,541 pregnancies, 13,988 were alive at 1 year. At age 7 years, this sample was bolstered with an additional 913 children. The total sample size for analyses using any data collected after the age of 7 is therefore 15,454 pregnancies; of these 14,901 were alive at 1 year of age. Participants are assessed at regular intervals using clinical interviews, self-report questionnaires, medical records, and physical examinations. Please note that the study website contains details of all the data that are available through a fully searchable data dictionary and variable search tool: http://www.bristol.ac.uk/alspac/researchers/our-data/. To avoid potential confounding due to relatedness, one sibling per set of multiple births was randomly selected to guarantee independence of participants. Similarly, individuals who were closely related to each other (defined as a *φ* > 0.2, as calculated in PLINK v1.90b) were identified and one individual per related pair was removed at random. In total 75 individuals were removed due to relatedness. Furthermore, individuals that did not pass genotyping quality control checks (see supplementary note and Supplementary Figure S1 for more details) were removed (*N* = 203). A total of 8654 participants with genotypic data and at least one measure of a phenotypic measure remained eligible for analysis (Supplementary Figure S1).

### 2.2 | Ethics statement

The authors assert that all procedures contributing to this work comply with the ethical standards of the relevant national and institutional committees on human experimentation and with the Helsinki Declaration of 1975, as revised in 2008. Ethical approval for the study was obtained from the ALSPAC Ethics and Law Committee and the Local Research Ethics Committees (Bristol and Weston Health Authority: E1808 Children of the Nineties: ALSPAC, 28 November 1989 (for details see: www.bristol.ac.uk/alspac/researchers/research-ethics/).

Consent for biological samples was obtained in accordance with the Human Tissue Act (2004). Informed consent for the use of data collected via questionnaires and clinics was obtained from participants following the recommendations of the ALSPAC Ethics and Law Committee at the time. The main caregiver initially provided consent for child participation and from the age 16 years the offspring themselves have provided informed written consent.

### 2.3 | ED symptoms: behaviours

Excessive exercise, fasting for weight loss, binge eating, and purging were assessed at age 14, 16, and 18 years using adapted questionnaire items from the Youth Risk Behaviour Surveillance System (Kann et al., 1996), which has previously been validated in a population-based study (Field et al., 2004; Micali, De Stavola, et al., 2015; Micali, Solmi, et al., 2015). See Supplementary Figure S2 for a timeline of when data on behaviours was collected. The questions asked pertained the presence of these behaviours anytime during the previous year.

#### Excessive exercise.

Individuals who endorsed exercising for weight loss purposes and felt guilty about missing exercise and found it hard to meet other obligations, such as schoolwork, because of their exercise regime at any of the ages assessed (14, 16, or 18 years) were coded as engaging in excessive exercise.

#### Fasting for weight loss.

Participants reported the frequency of fasting (i.e., not eating for an entire day) to lose or avoid gaining weight during the past year. Similar to the excessive exercise variable, individuals that indicated any fasting for weight loss at any age (14, 16, or 18 years) were coded as engaging in fasting for weight loss.

#### Binge eating.

Participants reported the frequency of binge eating during the past year—defined as eating an unusually large amount of food, combined with a feeling of being out of control. Those who reported having engaged in binge eating at either age 14, 16, or 18 years were coded as engaging in binge eating.

#### Purging.

Participants reported the frequency with which they used laxatives or self-induced vomiting, or other medicines to lose or avoid gaining weight during the past year; individuals that responded affirmatively to all of these questions at either the age 14, 16, or 18 years were coded as engaging in purging.

### 2.4 | ED symptoms: cognitions

Fear of weight gain and weight and shape concern were also assessed with adapted questionnaire items from the Youth Risk Behaviour Surveillance System (Kann et al., 1996). See Supplementary Figure S2 for a timeline of when data on cognitions was collected.

#### Fear of weight gain.

At age 13, 14 and 16 years, parents reported if, at any time in the previous year, their child was afraid of gaining weight or getting fat (0 = ‘No’, 1 = ‘A little’, 2 = ‘A lot’, and 3 = ‘It really terrified him/her’), if they avoided fattening foods (0 = ‘No’, 1 = ‘A little’, 2 = ‘A lot’) or whether their child felt it would be difficult or impossible if they were asked to put on 2 kilos for the sake of their health (0 = ‘Easy’, 1 = ‘Difficult’, 2 = ‘Impossible’). The maximum value of either of the three ages (13, 14, and 16 years) was used for the analyses; a higher score indicates greater fear of weight gain.

#### Thin ideal internalisation.

At age 14 years, thin ideal internalisation was assessed using the Ideal-Body Stereotype Scale-Revised (Stice, 1998) with gender-specific items used to assess different aspects of appearance-ideal internalisation at any time in the previous year for girls and boys separately. Adolescent girls and boys reported how strongly they agreed a series of questions; for example, thin women being not attractive and thin men being more good-looking. The responses were rated on a five-point Likert scale from ‘strongly agree’ to ‘strongly disagree’ and the items from this scale were summed to obtain a total score; a higher score corresponded with increased internalisation of the thin ideal.

#### Weight and shape concern.

At age 14 years, weight and shape concerns were assessed by using questions from the McKnight Risk Factor Survey (Shisslak et al., 1999). Participants were asked how they felt about (i) the way their body looked, (ii) how much their weight had made a difference to how they feel about themselves, and (iii) how much they had worried about gaining a little weight (e.g., 1 kg) at any time in the previous year. Responses were captured on a four response Likert scale ranging from ‘very happy’ or ‘not at all’ to ‘very unhappy’ or ‘a lot’. These three questions were summed to generate an overall score.

#### Body dissatisfaction,

Body dissatisfaction was assessed at age 14 years using the Body Dissatisfaction Scale (Calzo et al., 2018; Micali, De Stavola, et al., 2015; Stice, 2001). Participants were asked gender-specific questions rating their satisfaction with nine body parts with responses on a six-point Likert scale ranging from ‘extremely satisfied’ to ‘extremely dissatisfied’ at any time in the previous year. We constructed a continuous score from this scale with a higher score corresponding to higher body dissatisfaction.

### 2.5 | Body mass index

BMI (weight/height^2^) was calculated using objectively measured weight and height obtained during a face-to-face assessment at age 11 years (Boyd et al., 2013, 2019; Fraser et al., 2013; Northstone et al., 2019). Height was measured to the nearest millimetre using a Harpenden Stadiometer (Holtain Ltd., Crymych, UK) and weight was measured using the Tanita Body Fat analyser (Tanita TBF UK Ltd., Middlesex, UK) to the nearest 50 g. Age- and sex-standardized BMI z-scores (zBMI) were calculated according to UK reference data, indicating the degree to which a child is heavier (>0) or lighter than expected according to his/her age and sex (Cole et al., 2000).

### 2.6 | Selection of PGS

PGSs index an individual’s genetic liability to a trait. They are calculated by summing the genetic variants an individual carries and weighting these genetic variants by the corresponding effect sizes that have been detected in a previous independent GWASs. Using GWAS summary statistics from independent samples, we calculated PGSs for 25 traits that we grouped into three broad categories; (i) PGS from psychiatric/neurological/behavioural traits and one PGS indexing educational attainment (*N*_PGS_ = 16); (ii) metabolic traits (*N*_PGS_ = 4); and (iii) anthropometric traits (*N*_PGS_ = 5). In the first category, the selection of the psychiatric PGSs was partially based on the previously reported significant genetic correlations of AN with these traits (Watson et al., 2019): schizophrenia (Ripke et al., 2014), post-traumatic stress disorder (Duncan et al., 2018), obsessive–compulsive disorder (OCD; Arnold et al., 2018), neuroticism (Hübel et al., 2019), major depressive disorder (Wray et al., 2018), and lifetime probable anxiety disorder (Purves et al., 2020). To test the hypothesis whether ED symptoms are related to a broader array of psychiatric traits we also included PGSs for lifetime cannabis use (Pasman et al., 2018), insomnia (Jansen et al., 2019), borderline personality disorder (Witt et al., 2017), bipolar disorder (Stahl et al., 2019), autism spectrum disorder (Grove et al., 2019), attention deficit hyperactivity disorder (ADHD; Demontis et al., 2019), AN (Watson et al., 2019), and alcohol dependence (Walters et al., 2018). We also calculated PGS for physical activity (Hübel et al., 2019), migraine (Gormley et al., 2016), and educational attainment (Lee et al., 2018) as previous research suggests an association with AN (Watson et al., 2019). In the second category, the selection of metabolic traits was also partially based on the previously identified genetic correlations with AN (Watson et al., 2019): type 2 diabetes (Scott et al., 2017), insulin resistance (Scott et al., 2017), fasting insulin (Dupuis et al., 2010), and high-density lipoprotein (HDL) cholesterol (Teslovich et al., 2010). Lastly, the third category included the anthropometric traits of height (Yengo et al., 2018), fat-free mass, fat mass, and body fat percentage (Hübel et al., 2019); all of these traits were previously associated with either AN or ED symptoms (Hübel et al., 2019; Watson et al., 2019). For a full list of PGS included in this study, see Supplementary Table S1.

### 2.7 | Polygenic risk scoring

We used PRSice version 2.2.3 for calculating the PGSs (Choi & O’Reilly, 2019). We clumped the SNPs that were present both in the summary statistics of the trait and in the genotype data of the ALSPAC (i.e., overlapping SNPs) to obtain genetically independent SNPs. We retained the SNP with the smallest *p* value in each 250 kilobase window of all those in linkage disequilibrium (*r*^*2*^ > 0.1). We calculated PGS at their optimal *p* value threshold in each individual by scoring the number of effect alleles (weighted by the allele effect size) across the set of remaining SNPs. We calculated the PGS using the high-resolution scoring (i.e., incrementally across a large number of *p* value thresholds) method to identify the *p* value threshold at which the PGS is optimally associated with the outcome and explains the most variance (i.e., resulting in the highest adjusted *R*^2^ for continuous outcomes and Nagelkerke’s *R*^2^ on the liability scale for binary outcomes). We evaluated the associations between PGS and ED symptoms using generalized linear models, adjusted for sex and the first six principal components. To adjust for overfitting, we permuted case-control status at every *p* value threshold 10,000 times and calculated empirical *p* values. For the associations between the PGS and the binary ED symptoms (i.e., binge eating, excessive exercise, fasting, fear of weight gain, and purging) we report odds ratios (ORs) and for the associations between the polygenic scores and the continuous ED symptoms (body dissatisfaction, thin ideal internalisation, and weight and shape concerns) we report beta coefficients. To correct for multiple testing (i.e., 25 PGS regression models), we calculated *Q* values using the false discovery rate approach (Benjamini & Hochberg, 1995).

### 2.8 | Exploratory causal mediation analyses

In previous work, we demonstrated that BMI is associated with ED symptoms in ALSPAC (Abdulkadir et al., 2020) and given reports that psychiatric disorders and educational attainment are associated with BMI (Benson et al., 2018; Hübel et al., 2019; McCrea et al., 2012), it is therefore possible that association between psychiatric and the education-related PGS with an ED symptoms could be mediated through measured BMI. As a first step, in the case of a significant association in the main PGS analyses (described above), the psychiatric and the education-related PGS were tested for an association with BMI at age 11 years (before endorsement of the ED symptoms). Only the PGS that showed both a significant association with an ED symptom and BMI at age 11 years were included in the mediation analyses to estimate the natural direct and indirect effect using concepts proposed in modern causal inference (VanderWeele, 2015). The natural direct effect (also known as the ‘average direct effect’) measures the mean or expected risk difference (in the case of a binary outcome measure) had the PGS been hypothetically set to change by 1 SD from 0 to 1, while at the same time childhood BMI had been set to take its natural value (i.e., the value that would be experienced had the PGS been set at the reference value of 0). The natural indirect effect (also known as the average causal mediation effect) measures the expected mean difference or expected risk difference (in the case of a binary outcome measure) had the PGS been hypothetically set to take the value 1, while at the same time childhood BMI had been set to take its potential values had PGS been set to 0 or 1. When summed, these direct and indirect effects give the total causal effects and therefore are useful measures of the contribution made by a particular pathway to a causal relationship. The mediation analyses were carried out using the ‘mediate’ package in R (Tingley et al., 2014). Analyses were controlled for biological sex, and the first six ancestry-informative principal components. Furthermore, we assumed that there were no additional unaccounted confounders nor any intermediate confounders (VanderWeele, 2015).

## 3 | RESULTS

### Sample description.

**FIGURE 1:**
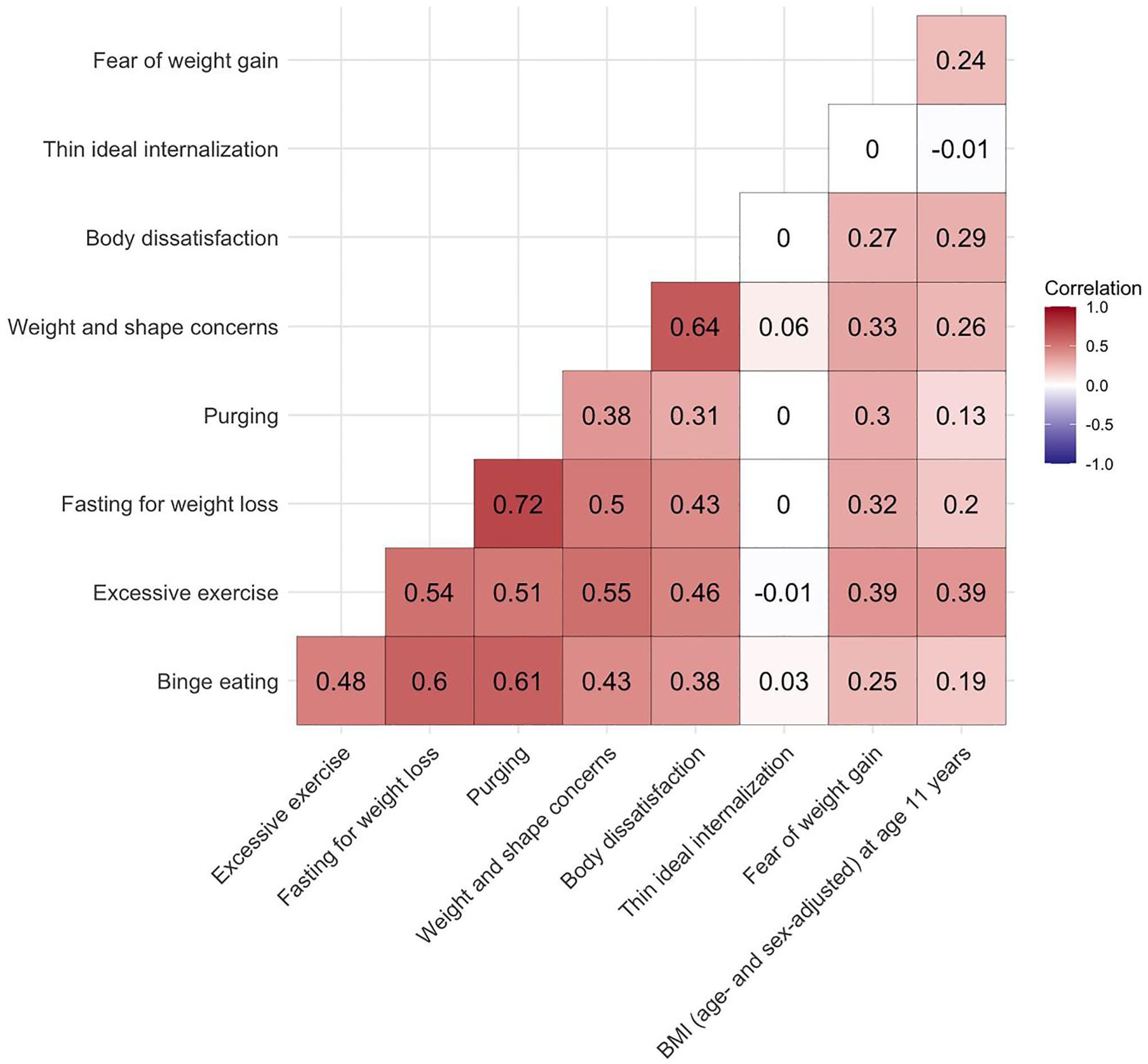
Heterogeneous correlation matrix of the investigated eating disorder (ED) symptoms and body mass index (BMI) in the Avon Longitudinal Study of Parents and Children (ALSPAC) (Boyd et al., 2013; Fraser et al., 2013; Golding, 2004) using the R package ‘polycor’ (version 0.7-10). Sample sizes per outcome were binge eating (*n* cases = 840, *n* controls = 2151), excessive exercise (*n* cases = 2,332, *n* controls = 2893), fasting for weight loss (*n* cases = 777, *n* controls = 2678), fear of weight gain (*n* = 6,013, mean = 1.62, SD = 1.24), purging (*n* cases = 398, *n* controls = 2962), body dissatisfaction (*n* = 4,624, mean = 21.85, SD = 7.75), thin ideal internalisation (*n* = 4,495, mean = 15.33, SD = 2.69), weight and shape concerns (*n* = 4,622, mean = 5.34, SD = 1.85), zBMI at age 11 years (*n* = 4,219, mean = 0.27, SD = 1.16). SD = standard deviation

Following quality control of the genetic data, between 2991 and 5225 individuals remained eligible for analyses with both genotype and phenotype data (Tables 1 and 2). We observed weak to moderate correlations (*r* = 0.13–0.72; Figure 1) among the ED symptom variables with a notable exception for thin ideal internalisation that showed negligible correlations with any of the ED symptoms (*r* = −0.01–0.06). Thin idealisation also differed from the other ED symptoms as it was the only ED symptom that showed consistent negative correlation with BMI at age 11 years.

### Polygenic scores associated with ED symptoms.

**FIGURE 2:**
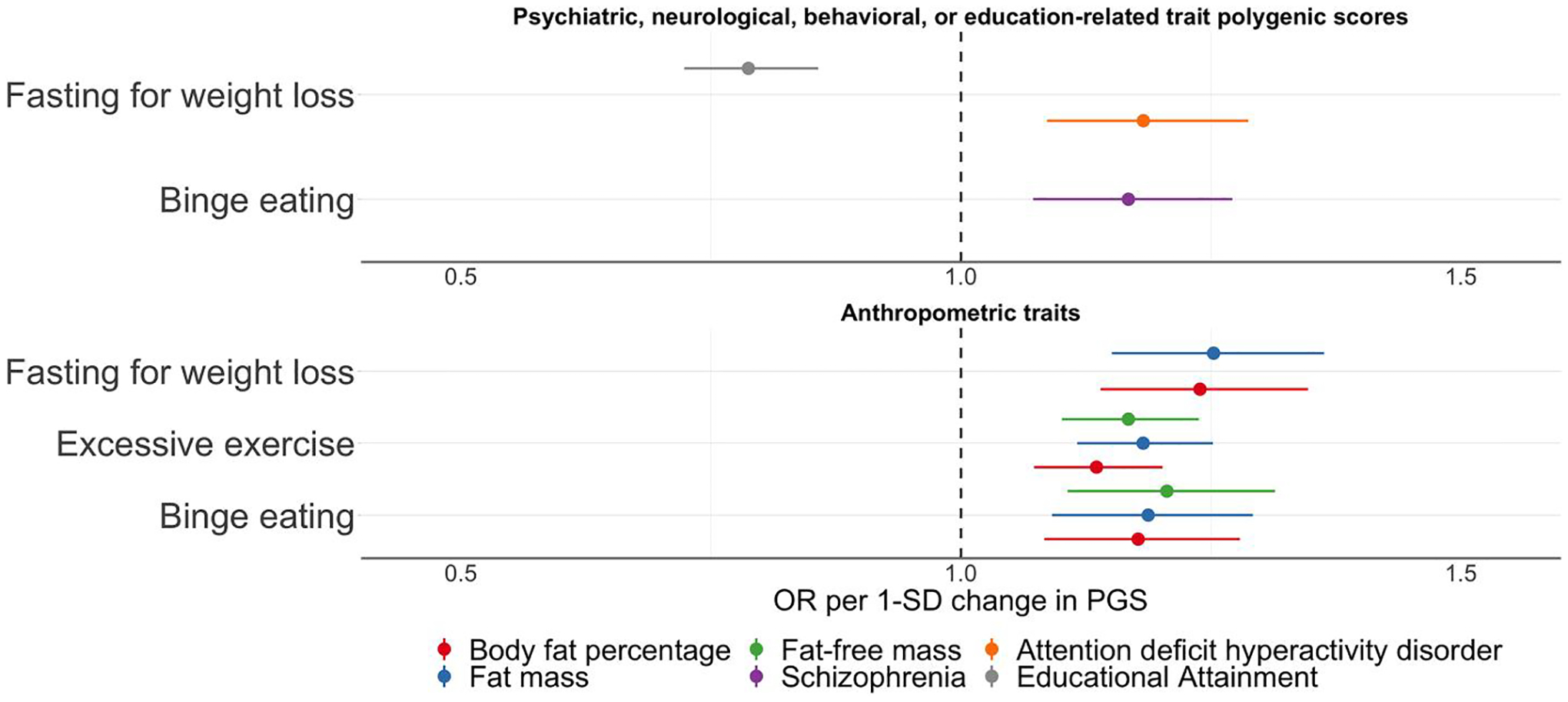
Polygenic scores associated with binge eating, excessive exercise, and fasting for weight loss in the Avon Longitudinal Study of Parents and Children. Sample sizes per outcome were binge eating (*n* cases = 840, *n* controls = 2151), excessive exercise (*n* cases = 2,332, *n* controls = 2893), fasting for weight loss (*n* cases = 777, *n* controls = 2678). Polygenic scores were calculated using PRSice v2 (Choi & O’Reilly, 2019). The optimal *p* value threshold to generate the polygenic score was obtained by calculating polygenic scores across multiple thresholds and permuting case-control status at each threshold 10,000 times. The polygenic score explaining the largest trait variance was used in logistic regressions including the first six ancestry principal components and sex as covariates. Shown here are only the associations between the polygenic scores and the binary eating disorder symptoms that remained significant after correction for multiple testing via calculation of false discovery rate-adjusted *Q* values (refer to Supplementary Tables S2 and S3 for a complete overview of the results). The dots represent the point estimates of the odd ratios for an increase of one standard deviation in the polygenic score and the lines represent the 95% confidence interval of the point estimate

**FIGURE 3:**
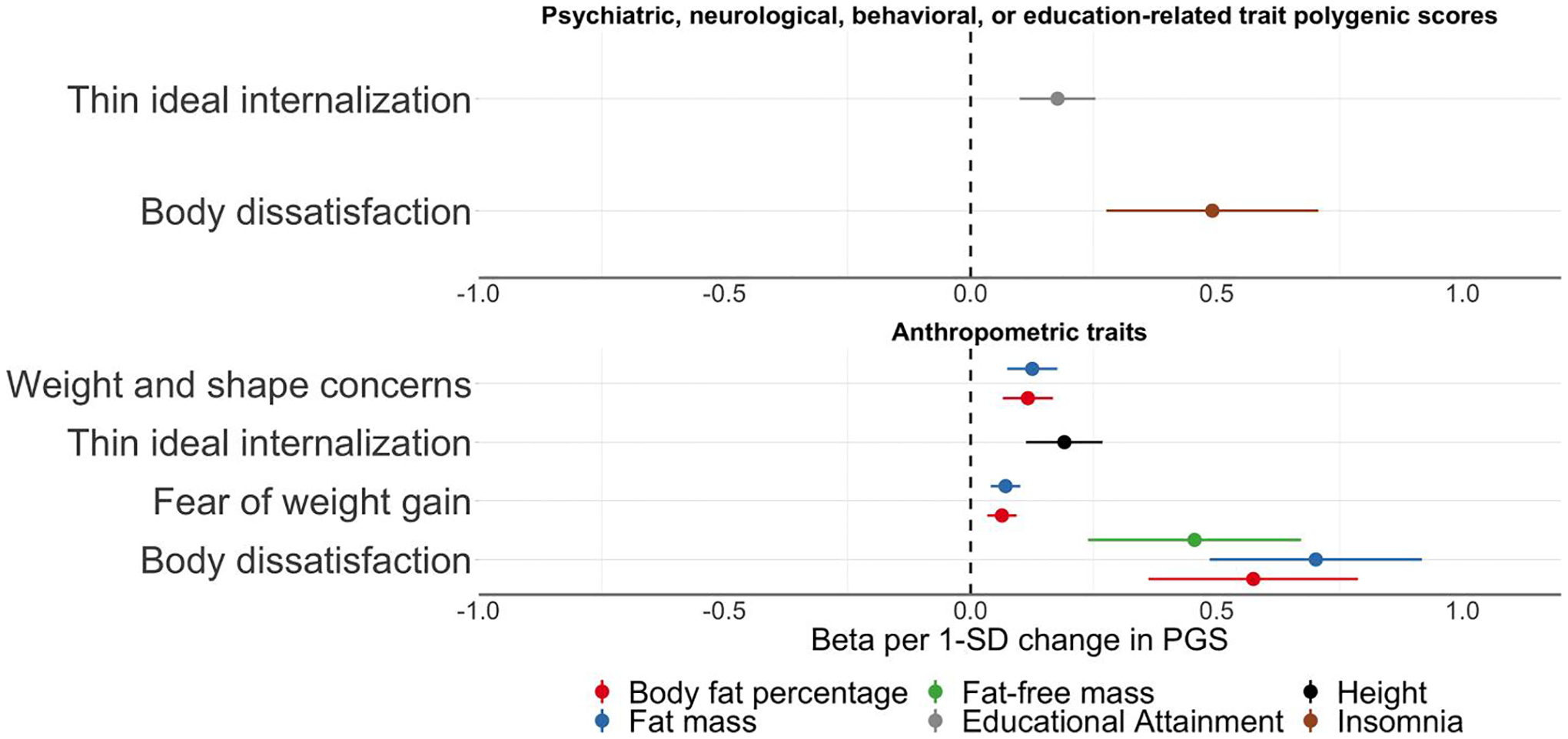
Polygenic scores associated with body dissatisfaction, fear of weight gain, thin ideal internalisation, and weight and shape concerns in the Avon Longitudinal Study of Parents and Children. Sample sizes per outcome were body dissatisfaction (*n* = 4,624, *mean* = 21.85, *SD* = 7.75), fear of weight gain (*n* = 6,013, *mean* = 1.62, *SD* = 1.24), thin ideal internalisation (*n* = 4,495, *mean* = 15.33, *SD* = 2.69), and weight and shape concerns (*n* = 4,622, *mean* = 5.34, *SD* = 1.85). Polygenic scores were calculated using PRSice v2. The optimal *p* value threshold to generate the polygenic score was obtained by calculating polygenic scores across multiple thresholds and permuting case-control status at each threshold 10,000 times. The polygenic score explaining the largest trait variance was used in linear regressions including the first six ancestry principal components and sex as covariates. Shown here are only the associations between the polygenic scores and the continuous eating disorder symptoms that remained significant after correction for multiple testing via calculation of false discovery rate-adjusted *Q* values (refer to Supplementary Tables S2 and S3 for a complete overview of the results). The dots represent the point estimates of the β for an increase of one standard deviation in the PGS and the lines represent the 95% confidence interval of the point estimate

After correcting for multiple testing using the false discovery rate adjustment, eight PGS were significantly associated with ED symptoms in ALSPAC. Reported are odds ratios per one standard deviation increase in the PGS for the binary ED symptoms (i.e., binge eating, excessive exercise, fasting for weight loss, and purging) and betas per one standard deviation increase in the PGS for the continuous ED symptoms (i.e., body dissatisfaction, thin ideal internalisation, weight and shape concerns, and fear of weight gain); Table 3, Figures 2–3, and Supplementary Tables S2 and S3.

### Association with psychiatric, neurological, behavioural, or education-related traits PGSs.

Four PGSs reflecting polygenic liability to psychiatric disorders or an education-related trait showed significant associations with ED symptoms (Table 3, Figures 2 and 3). The schizophrenia PGS was positively associated with binge eating (OR per standard deviation in PGS = 1.17, 95% confidence interval [CI]: 1.07, 1.27; *Q* = 0.04). Higher insomnia PGS was positively associated with body dissatisfaction (*β* = 0.49, 95% CI: 0.28, 0.71; *Q* < 0.01). Individuals with higher educational attainment PGS were less likely to engage in fasting behaviour (OR = 0.79, 95% CI: 0.72, 0.86; *Q* < 0.01), and had on average higher scores on thin ideal internalisation compared to individuals with lower PGS on educational attainment (*β* = 0.18, 95% CI: 0.10, 0.25; *Q* < 0.01). Additionally, individuals with one standard deviation higher ADHD PGS were more likely to engage in fasting behaviour (OR = 1.18, 95% CI: 1.09, 1.29; *Q* = 0.03). We found no evidence of association for the psychiatric, neurological, behavioural, or education-related trait PGSs with excessive exercise and weight and shape concerns.

### Association with metabolic trait PGSs.

None of the PGSs indexing metabolic traits were associated with any ED symptoms, after correcting for multiple comparisons (Supplementary Tables S2 and S3).

### Association with anthropometric trait PGSs.

Several PGSs of anthropometric traits were positively associated with ED symptoms (Table 3, Figures 2 and 3, and Supplementary Tables S2 and S3). For instance, the height PGS was positively associated with thin ideal internalisation (*β* = 0.19, 95% CI: 0.11, 0.27; *Q* < 0.01), whereas the fat-free mass PGS was positively associated with binge eating (OR = 1.21, 95% CI: 1.11, 1.31; *Q* < 0.01), excessive exercise (OR = 1.17, 95% CI: 1.10, 1.24; *Q* < 0.01), and body dissatisfaction (*β* = 0.46, 95% CI: 0.24, 0.67; *Q* < 0.01). A similar pattern was observed for the fat mass PGS which was positively associated with binge eating (OR = 1.19, 95% CI: 1.09, 1.29; *Q* = 0.01), excessive exercise (OR = 1.18, 95% CI: 1.12, 1.25; *Q* < 0.01), fasting (OR = 1.25, 95% CI: 1.15, 1.36; *Q* < 0.01), body dissatisfaction (*β* = 0.70, 95% CI: 0.49, 0.92; *Q* < 0.01), and weight and shape concerns (*β* = 0.13, 95% CI: 0.07, 0.18; *Q* < 0.01). The body fat percentage PGS showed the same pattern as the fat mass PGS; that is, a positive association with binge eating (OR = 1.18, 95% CI: 1.08, 1.28; *Q* = 0.02), excessive exercise (OR = 1.14, 95% CI: 1.07, 1.20; *Q* < 0.01), fasting (OR = 1.24, 95% CI: 1.14, 1.35; *Q* < 0.01), body dissatisfaction (*β* = 0.57, 95% CI: 0.36, 0.79; *Q* < 0.01), weight and shape concerns (*β* = 0.12, 95% CI: 0.07, 0.17; *Q* < 0.01). The similarity of the results is likely due to either phenotypic correlations (e.g., fasting for weight loss and binge eating *r* = 0.60; Figure 1), correlation between the PGSs (fat mass and body fat percentage *r* = 0.89; Supplemental Figure S3), or a combination of both.

### Exploratory causal mediation analyses.

Prior to inclusion in the mediation analyses, we tested whether the ADHD, educational attainment, insomnia, and the schizophrenia PGS were associated with BMI at age 11 years (Supplementary Table S4); only the ADHD (*β* = 0.13, 95% CI: 0.06, 0.20; *Q* < 0.01) and the educational attainment PGS (*β* = −0.19, 95% CI: −0.26, −0.12; *Q* < 0.01) were significantly associated with BMI at age 11 years and were therefore included in the causal mediation analyses. Childhood BMI significantly mediated the association between the ADHD PGS and fasting for weight loss (*β* average causal mediation effect = 0.004, 95% CI: 0.002, 0.007; *p* < 0.01); one SD increase in the ADHD PGS corresponded with a higher BMI at age 11 years, which in turn increased the probability of endorsing fasting for weight loss. Furthermore, we found that BMI significantly mediated the association between the educational attainment PGS and fasting for weight loss (*β* average causal mediation effect = −0.004, 95% CI: −0.006, −0.002; *p* < 0.01; Table 4); that is, a one standard deviation increase in the educational attainment PGS corresponded with a decrease in BMI at age 11 years, which in turn led to a decreased probability of endorsing fasting for weight loss during adolescence. However, we did not observe a significant mediation effect by BMI in the association between the educational attainment PGS and thin ideal internalisation (*β* average causal mediation effect = −0.002, 95% CI: −0.005, 0.001; *p* = 0.27).

## 4 | DISCUSSION

We report molecular genetic evidence that genetics of psychiatric disorders and anthropometric traits, but not metabolic traits, as indexed by PGSs are associated with ED symptoms and traits present in a general population cohort in adolescence. Notably, several ED symptoms (i.e., binge eating, fasting for weight loss, and body dissatisfaction) in this study were significantly associated with both psychiatric and anthropometric PGSs suggestion shared genomics and etiological contributions. Importantly, contrary to our hypotheses, and unlike previously published results for AN (Duncan et al., 2017; Watson et al., 2019), ED symptoms were not associated with the selected metabolic PGSs (i.e., type 2 diabetes, insulin resistance, fasting insulin, and high-density lipoprotein [HDL] cholesterol).This pattern suggests that ED symptoms in the population and threshold EDs may be partially etiologically related (i.e., psychiatric and anthropometric origins), but that metabolic genetic factors may differentiate between symptoms and threshold EDs.

Our results highlight novel associations between educational attainment and ED symptoms. An increase in educational attainment PGS was associated with lower likelihood of endorsing fasting for weight loss and higher levels of thin ideal internalisation (the extent to which a person agrees with socially defined ideals of attractiveness, such as the notion that thin individuals are attractive). In previous work, we demonstrated that fasting for weight loss was positively associated with BMI (Abdulkadir et al., 2020) and given reports of a causal relationship between higher education and decreased fat mass (Hübel et al., 2019), it is not surprizing that the educational attainment PGS was negatively associated with fasting for weight loss. Further exploration of the association between the educational attainment PGS and fasting for weight loss revealed that this association is mediated through BMI; an increase in educational attainment PGS leads to a decrease in childhood BMI which in turn leads to a lower probability of endorsing fasting for weight loss during adolescence. Furthermore, the positive association between higher educational attainment PGS and thin ideal internalisation is consistent with several studies that reported positive genetic correlation (*r*_g_ = 0.25) between educational attainment and AN (Duncan et al., 2017; Watson et al., 2019); our measure of thin ideal internalisation captures body image disturbances that are considered a core component of AN. Interestingly, in contrast to our finding for fasting for weight loss, the association between the educational attainment PGS and thin ideal internalisation was not mediated through BMI. Taken together, our findings suggest that thin ideal internalisation as measured in the general population could share the same genetic risk as AN in part through alleles that influence educational attainment independent of BMI.

Another novel finding to emerge from our study is the positive association between the insomnia PGS and body dissatisfaction; a finding that aligns with previous reports (Akram, 2017; Gupta et al., 2015) of positive associations between self-reported insomnia symptoms and dissatisfaction with cutaneous body image (mental perception of the appearance of the skin, hair, and nails). We sought to further understand the association between the insomnia PGS and body dissatisfaction by testing whether BMI is a possible mediator; body dissatisfaction was associated with BMI in this sample (Abdulkadir et al., 2020) and could therefore operate as a mediator. Counter to our expectation, we found no association between the insomnia PGS and childhood BMI suggesting that childhood BMI is likely not on the causal pathway between the insomnia PGS and body dissatisfaction in adolescence.

Returning to the absence of a significant association between metabolic PGSs and ED symptoms, alternative interpretations exist. First, the finding might be true, and the absence of metabolic effects could represent a point of divergence between ED symptoms and threshold disorders. Alternatively, the majority of ED symptoms studied here (apart from thin ideal internalisation and fear of weight gain) are more closely related to bulimic type disorders, and no GWAS of binge-type eating disorders have yet been conducted. The metabolic PGSs that we selected were based on significant correlations observed in an AN GWAS. Emerging bulimia and binge-eating GWAS may implicate different metabolic factors that were not captured here. If the result holds, genetically influenced metabolic vulnerability may represent the ‘tipping point’ between subthreshold symptoms and threshold disorders. This notion aligns with findings from studies that suggest that AN might be more distinctly genetically demarcated from ED symptoms in the general population than other EDs (Adams et al., 2020; Dinkler et al., 2019).

Purging stood out in our analyses as it was not associated with any of the 25 PGSs. It is likely that our analyses were underpowered for purging as this trait was the least frequently endorsed in our study population (*N* case = 398).

Our findings must be interpreted in light of the following limitations. The ED symptoms were self-reported, and the questions pertained to anytime in the previous year, which for some participants may have caused recall bias if the symptoms occurred further away from the time at assessment. Some of the ED symptoms (i.e., fasting for weight loss and fear of weight gain) were defined using a single item which might not necessarily capture the phenotype accurately. Ideally, clinician-assessed measures are preferred, but this is not feasible for large cohort studies, such as ALSPAC. We did not have information on ED symptoms in late childhood; occurrence of these behaviours prior to the measurement of BMI at age 11 years could have biased estimates of our mediation analysis. However, it is likely that prevalence of these behaviours and cognitions would have been very low in late childhood. Furthermore, the sample only included white British participants and therefore the results of this study are not generalisable to other populations. Like many other longitudinal studies, in the ALSPAC study, participants tend to drop out as time goes on leading to missing data. However, we would like to emphasize that the longitudinal nature of the study allowed us to define phenotypes across different time-points, which in part helped in mitigating missingness. Furthermore, there is evidence that indicates that higher PGSs on smoking initiation, schizophrenia, and depression in ALSPAC participants are associated with higher dropout rates from the study (Taylor et al., 2018); therefore, any bias introduced by higher dropout likely attenuated associations towards the null.

The associations between ED symptoms, that are widely prevalent in the community (Nadia Micali et al., 2014), and psychiatric and anthropometric PGSs highlight that these symptoms are polygenic and may share a genetic architecture with AN but yet could differ from AN in one important component, namely metabolic disturbances. It is critical to understand why some individuals who endorse ED symptoms progress to develop threshold AN whereas others do not; metabolic disturbances could be the catalyst that puts some individuals on a developmental trajectory leading to AN. Future studies are needed to understand when during development these metabolic disturbances manifest and how they may affect developmental trajectory of AN. A better understanding of this process could bring us closer to improved therapeutics for AN. Furthermore, findings from out mediation analysis show that some of the associations found between the PGSs and ED symptoms are mediated through BMI suggesting that targeting childhood BMI and psychopathology (e.g., ADHD) might aid in the early detection or the prevention of ED symptoms in puberty. Our findings also point to the need of further exploring the overlap between risk for psychiatric disorders and anthropometry when aiming to understand the development of ED. However, given the effect sizes of PGS at the current stage, the clinical utility of PGS is premature.

In conclusion, our results suggest that ED symptoms as present in the general population are genetically associated with psychiatric and anthropometric traits but not with metabolic traits. Our findings serve as a base for future translational research aimed at understanding the potential shared biological mechanisms among ED symptoms, psychiatric traits, and anthropometric traits, and differences in risk for ED symptoms versus threshold EDs, as implied by the findings of this study.

## Supplementary Material

Supplementary_note_and_figures

Supplementary_tables

## Figures and Tables

**TABLE 1:** Descriptive statistics of the binary eating disorder (ED) symptoms outcomes of the participants in the Avon Longitudinal Study of Parents and Children^a^

Eating disorder symptom	Age phenotype measured	Total *N*	Cases *N* (%)	Cases (% female)	Controls *N*	Controls (% female)
Binge eating	14, 16, 18	2991	840 (28.08)	78.81	2151	54.3
Excessive exercise	14, 16, 18	5225	2332 (44.63)	72.14	2893	42.62
Fasting for weight loss	14, 16, 18	3455	777 (22.49)	87.52	2678	53.02
Purging	14, 16, 18	3360	398 (11.85)	88.94	2962	56.25

a Full description of the Avon Longitudinal Study of Parents and Children is described elsewhere (Boyd et al., 2013; Fraser et al., 2013; Golding, 2004). The descriptive depicted in this table are on individuals for which genotype information was available.

**TABLE 2:** Descriptive statistics of the continuous eating disorder (ED) symptoms outcomes and body mass of the participants in the Avon Longitudinal Study of Parents and Children^a^

Continuous measures	Total *N*	Mean (SD)	Observed range (min, max)
Body dissatisfaction^b^	4624	21.85 (7.75)	9, 46.3
Thin ideal internalisation^b^	445	15.33 (2.69)	5, 25
Weight and shape concerns^b^	4622	5.34 (1.85)	3, 12
Fear of weight gain^b^	6013	1.62 (1.24)	0, 7
Age- and sex-adjusted body mass index (zBMI)^c^	4219	0.27 (1.16)	−4.9, 3.6

Abbreviation: SD, standard deviation.

a Full description of the Avon Longitudinal Study of Parents and Children is described elsewhere (Boyd et al., 2013; Fraser et al., 2013; Golding, 2004). The descriptive depicted in this table are on individuals for which genotype information was available.

b Symptoms were all measured at age 14 years except for fear of weight gain which was measured at age 13, 14, and 16 (the maximum value of either of the three ages was used for the analyses).

c Calculated as weight in kilograms divided by height in metres squared. Height was measured to the nearest millimetre using a Harpenden Stadiometer (Holtain Ltd., Crymych, UK) and weight was measured using the Tanita Body Fat analyser (Tanita TBF UK Ltd., Middlesex, UK) to the nearest 50 g at age 11 years zBMI was calculated by standardising BMI by age and sex.

**TABLE 3:** Associations of psychiatric, metabolic, and anthropometric polygenic scores (PGSs) with eating disorder (ED) symptoms

Psychiatric, neurological, behavioural, or education-related trait polygenic scores	Eating disorder symptom	Threshold^a^	*n* SNPs^a^	Or/*β* (95% CI)^b^	*Q* ^c^
Attention deficit hyperactivity disorder	Fasting for weight loss	0.0824001	21,419	1.18 (1.09,1.29)^d^	0.02
Educational attainment	Fasting for weight loss	0.0178001	18,377	0.79 (0.72,0.86)^d^	<0.01
Educational attainment	Thin ideal internalisation	0.00975005	13,501	0.18 (0.1,0.25)^e^	<0.01
Insomnia	Body dissatisfaction	0.0344501	16,542	0.49 (0.28,0.71)^e^	<0.01
Schizophrenia	Binge eating	0.3677	99,152	1.17 (1.07,1.27)^d^	0.04
Anthropometric traits polygenic scores	Eating disorder symptom	Threshold^a^	*n* SNPs^a^	OR/*β* (95% CI)^b^	*Q* ^c^
Body fat percentage	Binge eating	0.00000005	70	1.18 (1.08,1.28)^d^	0.02
Body fat percentage	Body dissatisfaction	0.00475005	5137	0.57 (0.36,0.79)^e^	<0.01
Body fat percentage	Excessive exercise	0.00605005	5981	1.14 (1.07,1.20)^d^	<0.01
Body fat percentage	Fasting for weight loss	0.00525005	5491	1.24 (1.14,1.35)^d^	<0.01
Body fat percentage	Fear of weight gain	0.20945000	71,864	0.06 (0.03,0.09)^e^	<0.01
Body fat percentage	Weight and shape concerns	0.00000005	70	0.12 (0.07,0.17)^e^	<0.01
Fat-free mass	Binge eating	0.0471501	27,189	1.21 (1.11,1.31)^d^	<0.01
Fat-free mass	Body dissatisfaction	0.4226	118,834	0.46 (0.24,0.67)^e^	<0.01
Fat-free mass	Excessive exercise	0.05075010	28,550	1.17 (1.10,1.24)^d^	<0.01
Fat mass	Binge eating	0.00025005	1024	1.19 (1.09,1.29)^d^	0.01
Fat mass	Body dissatisfaction	0.0274001	16,718	0.7 (0.49,0.92)^e^	<0.01
Fat mass	Excessive exercise	0.05075010	29,031	1.18 (1.12,1.25)^d^	<0.01
Fat mass	Fasting for weight loss	0.0618001	29,739	1.25 (1.15,1.36)^d^	<0.01
Fat mass	Fear of weight gain	0.00000005	82	0.07 (0.04,0.1)^e^	<0.01
Fat mass	Weight and shape concerns	0.0207501	13,733	0.13 (0.07,0.18)^e^	<0.01
Height	Thin ideal internalisation	0^a^00410005	13,912	0.19 (0.11,0.27)^e^	<0.01

Abbreviations: *n*SNP, number of single nucleotide polymorphisms included in polygenic score; OR, odds ratio; *β*, Beta; CI, confidence interval, *Q*, false discovery rate-adjusted *p* value.

a Polygenic scores were calculated using PRSice v2 (Choi & O’Reilly, 2019). The optimal *p* value threshold to generate the polygenic score was obtained by calculating polygenic scores across multiple thresholds and permuting case-control status at each threshold 10,000 times. The polygenic score explaining the largest trait variance was used in a regression model including the first six ancestry principal components and sex as covariates.

b Odds ratios and betas reflect one standard deviation change in the standardized (to mean zero and standard deviation of one) PGS.

c *Q*-values are the false discovery rate (FDR) adjusted *p* values using the Benjamini & Hochberg method (Benjamini & Hochberg, 1995) correcting for the total number comparisons. Shown in this table are only the associations that remained significant after FDR correction. For a full list of the results see Supplementary Tables S2–S3.

d Depicted values are odds ratios.

e Depicted values are *β*.

**TABLE 4:** Causal mediation analysis of the attention deficit hyperactivity disorder and the educational attainment polygenic score scores and the eating disorder (ED) symptoms fasting for weight loss and thin ideal internalisation with the age- and sex-adjusted body mass index (zBMI) at age 11 years as mediator^a^

Independent variable	Mediator	Dependent variable	*β* Average direct effect (95% CI)	*p* (average direct effect)	*β* Average causal mediation effect (95% CI)	*p* Average causal mediation effects
Educational attainment polygenic score	zBMI at age 11 years	Fasting for weight loss	−0.02 (−0.03, −0.01)	<0.01	−0.004 (−0.006, −0.002)	<0.01
Attention deficit hyperactivity disorder polygenic score	zBMI at age 11 years	Fasting for weight loss	0.01 (0, 0.03)	0.048	0.004 (0.002, 0.007)	<0.01
Educational attainment polygenic score	zBMI at age 11 years	Thin ideal internalisation	0.06 (0.02, 0.09)	<0.01	−0.001 (−0.004, 0.002)	0.43

*Note*: *p V*alues for mediation were generated with bootstrapping methods.

Abbreviation: CI, confidence interval.

a Psychiatric and education-related polygenic scores that showed a significant association with an ED symptom in the main analyses (see Table 3) and a significant associated with the age- and sex-adjusted body mass index (zBMI) at age 11 years (see Supplementary Table S4) are included here in a mediation model to estimate natural direct and indirect effects (average causal mediation effect). Mediation analyses was carried out using the R package ‘mediation’ (version 4.4.6; 32) which is based on concepts proposed in modern causal inference (VanderWeele, 2015). Prior to the mediation analyses the PGS were standardized and the analysis was controlled for biological sex and the first six ancestry principal components.

## Data Availability

The data that support the findings of this study are available on request from the corresponding author. The data are not publicly available due to privacy or ethical restrictions.

## Highlights

- Several eating disorder symptoms (i.e., binge eating, fasting for weight loss, and body dissatisfaction) in this study were significantly associated with both psychiatric and anthropometric polygenic scores emphasising the genetic complexity of these traits.
- Eating disorder symptoms as present in the general population and threshold eating disorders may be partially etiologically related (i.e., psychiatric and anthropometric origins), but metabolic genetic factors may differentiate between symptoms and threshold eating disorders.
- Metabolic disturbances could therefore be the catalyst that puts some individuals on a developmental trajectory leading to threshold eating disorders.

## Abbreviations:

ADHD: Attention deficit hyperactivity disorder
ALSPAC: Avon Longitudinal Study of Parents and Children
AN: Anorexia nervosa
BMI: Body mass index
*β*: Beta
ED: Eating disorder
FDR: False discovery rate
GWAS: Genome-wide association study
*h* ^2^: Narrow sense heritability
HDL: High-density lipoprotein
OR: Odds ratio
PGS: Polygenic score
*r*_g_: Genetic correlation
SD: Standard deviation
SNP: Single nucleotide polymorphism
zBMI: Body mass index *z*-scores
