# Supplementary material for: Eating disorder symptoms and their associations with anthropometric and psychiatric polygenic scores
Source: Eur Eat Disord Rev. 2022 Feb 17;30(3):221–36. doi: 10.1002/erv.2889 (PMC9149768; doi:10.1002/erv.2889)
Supplement: Supplementary file 1 — Supplementary Material [file ERV-30-221-s001.docx]

**Supplementary Note**

**Quality control checks**

Individuals with disproportionate levels of individual missingness (i.e., >3%), insufficient sample replication (identity by descent < 0.1), biological sex mismatch, and non-European ancestry (as defined by multi-dimensional scaling using the HapMap Phase II, release 22, reference populations) were excluded. SNPs with a minor allele frequency (MAF) of < 1%, excessive missingness (i.e., call rate < 95%), or a departure from the Hardy–Weinberg equilibrium (*P* value < 5 x 10^-7^) were removed. Imputation was conducted with Impute3 using the HRC 1.0 as the reference panel (McCarthy et al., 2016) and phasing was carried out using ShapeIT (v2.r644). Finally, post-imputation quality control checks were performed; any SNPs with MAF less than 1%, Impute3 information quality metric of < 0.8, and not confirming to Hardy-Weinberg equilibrium (*P* < 5 × 10^-7^) were removed. After data cleaning, a total of 8,654 individuals (4,225 females and 4,429 males) and 4,054,653 SNPs remained eligible for analyses.

**Figure S1:** Flowchart of participating individuals of the Avon Longitudinal Study of Parents and Children (ALSPAC) study (Boyd et al., 2013; Fraser et al., 2013; Golding, 2004).

**Figure S2:** Timeline of when phenotypic data was collected in participating individuals of the Avon Longitudinal Study of Parents and Children (ALSPAC) (Boyd et al., 2013; Fraser et al., 2013; Golding, 2004).

**Figure S3:** Correlation matrix of the investigated polygenic scores in the Avon Longitudinal Study of Parents and Children (ALSPAC) (Boyd et al., 2013; Fraser et al., 2013; Golding, 2004). For more information regarding sources of the polygenic scores see Table S1.

**References**

Boyd, A., Golding, J., Macleod, J., Lawlor, D. A., Fraser, A., Henderson, J., … Smith, G. D. (2013). Cohort profile: The ’Children of the 90s’-The index offspring of the avon longitudinal study of parents and children. *International Journal of Epidemiology*, *42*(1), 111–127. https://doi.org/10.1093/ije/dys064

Fraser, A., Macdonald-wallis, C., Tilling, K., Boyd, A., Golding, J., Davey smith, G., … Lawlor, D. A. (2013). Cohort profile: The avon longitudinal study of parents and children: ALSPAC mothers cohort. *International Journal of Epidemiology*, *42*(1), 97–110. https://doi.org/10.1093/ije/dys066

Golding, J. (2004). The Avon Longitudinal Study of Parents and Children (ALSPAC)--study design and collaborative opportunities. *European Journal of Endocrinology*, U119–U123. https://doi.org/10.1530/eje.0.151u119

McCarthy, S., Das, S., Kretzschmar, W., Delaneau, O., Wood, A. R., Teumer, A., … Marchini, J. (2016). A reference panel of 64,976 haplotypes for genotype imputation. *Nature Genetics*, *48*(10), 1279–1283. https://doi.org/10.1038/ng.3643
